# Introducing the Newly Isolated Bacterium *Aneurinibacillus* sp. H1 as an Auspicious Thermophilic Producer of Various Polyhydroxyalkanoates (PHA) Copolymers–1. Isolation and Characterization of the Bacterium

**DOI:** 10.3390/polym12061235

**Published:** 2020-05-29

**Authors:** Iva Pernicova, Ivana Novackova, Petr Sedlacek, Xenie Kourilova, Michal Kalina, Adriana Kovalcik, Martin Koller, Jana Nebesarova, Vladislav Krzyzanek, Kamila Hrubanova, Jiri Masilko, Eva Slaninova, Stanislav Obruca

**Affiliations:** 1 Faculty of Chemistry, Brno University of Technology, Purkynova 118, 612 00 Brno, Czech Republic; xcpernicovai@fch.vut.cz (I.P.); xcnovackova@fch.vut.cz (I.N.); sedkacek-p@fch.vut.cz (P.S.); xckourilovax@fch.vut.cz (X.K.); kalina-m@fch.vut.cz (M.K.); kovalcik@fch.vut.cz (A.K.); masilko@fch.vut.cz (J.M.); xcslaninovae@fch.vut.cz (E.S.); 2 Office of Research and Management, c/o Institute of Chemistry, NAWI Graz, University of Graz, Heinrichstrasse 28/VI, 8010 Graz, Austria; martin.koller@uni-graz.at; 3 ARENA Arbeitsgemeinschaft für Ressourcenschonende & Nachhaltige Technologien, Inffeldgasse 21b, 8010 Graz, Austria; 4 Biology Centre, The Czech Academy of Sciences, v.v.i., Branisovska 31, 370 05 Ceske Budejovice, Czech Republic; nebe@paru.cas.cz; 5 Faculty of Science, University of South Bohemia, Branisovska 31, 370 05 Ceske Budejovice, Czech Republic; 6 Institute of Scientific Instruments of the Czech Academy of Sciences, v.v.i., Kralovopolska 147, 612 64 Brno, Czech Republic; krzyzanek@isibrno.cz (V.K.); hrubanova@isibrno.cz (K.H.)

**Keywords:** polyhydroxyalkanoates, thermophiles, *Aneurinibacillus* sp., P(3HB-*co*-4HB), P(3HB-*co*-3HV-*co*-4HB)

## Abstract

Extremophilic microorganisms are considered being very promising candidates for biotechnological production of various products including polyhydroxyalkanoates (PHA). The aim of this work was to evaluate the PHA production potential of a novel PHA-producing thermophilic Gram-positive isolate *Aneurinibacillus* sp. H1. This organism was capable of efficient conversion of glycerol into poly(3-hydroxybutyrate) (P3HB), the homopolyester of 3-hydroxybutyrate (3HB). In flasks experiment, under optimal cultivation temperature of 45 °C, the P3HB content in biomass and P3HB titers reached 55.31% of cell dry mass and 2.03 g/L, respectively. Further, the isolate was capable of biosynthesis of PHA copolymers and terpolymers containing high molar fractions of 3-hydroxyvalerate (3HV) and 4-hydroxybutyrate (4HB). Especially 4HB contents in PHA were very high (up to 91 mol %) when 1,4-butanediol was used as a substrate. Based on these results, it can be stated that *Aneurinibacillus* sp. H1 is a very promising candidate for production of PHA with tailored material properties.

## 1. Introduction

The term “thermophiles” is devoted to microorganisms revealing optimal growth temperature above 45 °C [1]. Actually, these microbes are ubiquitous since they can be isolated from a wide range of habitats from freshly fallen snow to pasteurized milk to geothermal areas like hot springs [2]. Currently, thermophiles attract attention of industrial biotechnologists since bioprocesses operated at elevated temperatures have a number of advantages, including a reduced risk of microbial contamination, lowered jeopardy by phage infection, improved solubility of substrates such as polysaccharide-rich resources, continuous and direct recovery of volatile metabolites from fermentation broth such as alcohols or volatile fatty acids (VFAs) and decreased cooling costs and demands for cooling water due to the higher temperature difference between bioreactor and the ambient air. On the other side, it should be pointed out that final energy balance of the process must be precisely evaluated at large scale to estimate whether operation of the process at elevated temperature can be economically feasible. Moreover, another aspect which should be taken into account is reduced solubility of oxygen under higher temperatures, which might disadvantage aerobic processes [1]. Professor Chen has recently defined the concept of “Next Generation Industrial Biotechnology” (NGIB), which is based on utilization of extremophilic microorganisms as producing units. Since extremophiles are capable of thriving under conditions which exclude most common mesophilic contaminants, NGIB can be principally operated under semi-sterile or even non-sterile conditions, which reduces costs of the process. Moreover, since NGIB processes are naturally robust against contamination, they can be run in continuous mode for long periods. Continuous processes are positive in terms of productivity and efficiency of the process due to the elimination of permanent unproductive times of revamping, which is needed in non-continuous processes for emptying and preparation of the bioreactor between individual cultivation batches [3].

Polyhydroxyalkanoates (PHA) are microbial polyesters which are accumulated by numerous prokaryotes in form of intracellular granules primarily as storage compounds; nevertheless, they also enhance robustness of the microbes against various stressors [4]. In most microorganisms, PHA accumulation is favored when external carbon substrate is present in excess, but nitrogen, phosphorous or other elements are lacking in parallel, which inhibits multiplication of cells [5]. PHA are very promising materials because they can be used as fully biodegradable and bioresource-based alternatives to petrochemical polymers since mechanical and technological properties of PHA are similar to some polymers produced from petrochemical resources. In fact, properties of PHA strongly depend upon their monomer composition. The most common and the best-studied member of the PHA family, namely poly(3-hydroxybutyrate) (P(3HB)), the homopolymer of 3-hydroxybutyrate (3HB), has a rather high melting temperature (T_m_) of about 180 °C, which is very close to the temperature of its degradation (T_d_; about 200 °C). It is remarkable that the native PHA material, organized as intracellular granules (“carbonosomes”) in the cytoplasm of bacterial cells, is completely amorphous, however, when the material is extracted from the cells, it quickly crystallizes; therefore, the material is rigid and brittle with only limited elongation at break (about 3%) [6]. Nevertheless, material properties can be tailored when other monomer subunits are incorporated into the polymer chain. For instance, when 3-hydroxyvalerate (3HV) is inserted into the polymer chain, the resulting copolymer poly(3-hydroxybuytyrate-*co*-3-hydroxyvalerate) (P(3HB-*co*-3HV)) possesses a lower melting temperature and also a less crystalline structure, which are very positive features considering the processing of the materials [7]. Alternatively, 4-hydroxybuytrate (4HB) can be also introduced into the polymer by some bacteria and haloarchaea; PHA containing 4HB subunits reveal only minor crystallinity, high flexibility and even improved biodegradability in numerous environments including the human body. Therefore, they are very suitable for applications in various fields including high-end medical applications such as implants or drug delivery systems [8]. 

To achieve biosynthesis of above mentioned copolymers, most microbes require supplementation of precursors structurally related to particular monomers. For instance, to induce synthesis of polymers containing 3HV subunits, odd carbon number precursors such as n-propanol, propionate, n-pentanol or valerate are applied. Similarly, for production of 4HB-containing polymers, precursor compounds structurally related to 4HB such as 1,4-butanediol, γ-butyrolactone (GBL) or 1,6-hexanediol are utilized [9].

PHA are very promising materials; however, they are disadvantaged in competition with petrochemical polymers by their high production costs [10]. There are several strategies which could facilitate sustainable and economically feasible production of PHA such as utilization of cheap carbon substrates, for instance waste or side products of food industry or agriculture [11], or the use of genetically engineered strains revealing high productivity and substrate-to-product conversion yields [12]. Furthermore, considering all the positive aspects of employing thermophiles in industrial biotechnology mentioned above, also the use of thermophilic PHA producers holds promise to gain sustainable and economically reasonable processes for PHA production. Nevertheless, despite the fact that PHA accumulation is a common feature among many prokaryotes, capability of PHA synthesis was described only for limited number of thermophiles such as *Chelatococcus thermostellatus* [13], *Thermus thermophilus* [14] or *Caldimonas taiwanensis* [15]. 

Therefore, we made an effort to isolate novel promising PHA-producing thermophiles. To reach this goal, we have recently developed a unique protocol for isolation of PHA-producing thermophiles described in [16]. We have isolated several interesting strains, and, among them, the isolate designated as H1 was identified to be the most promising one. Therefore, we have performed taxonomic classification, basic morphological and metabolic description and, what is the most important, we have also assessed the biotechnological potential of this novel thermophilic isolate for industrial production of PHA and various PHA copolymers with tailored material properties.

## 2. Materials and Methods

### 2.1. Isolation and Characterization of the Thermophilic PHA Producer 

**Figure 1:**
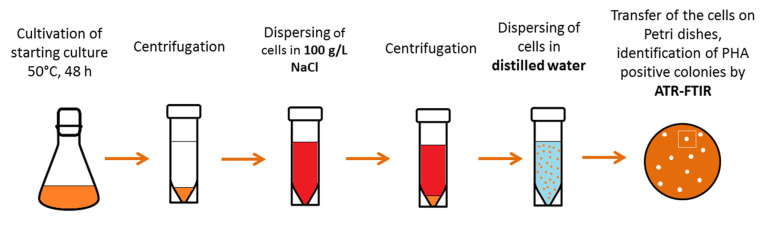
Principle of the “osmoselection”; procedure enabling isolation of PHA-producing thermophiles.

A compost sample was taken from the central urban composting plant of the city Brno, Czech Republic (Centrální kompostárna Brno operated by SUEZ CZ a.s.), the temperature of the compost was about 40 °C at time of sample collection. After homogenization of the compost collected, 1 g of the sample was mixed with 100 mL of mineral salt medium (MSM) of the following composition (g/L): Na_2_HPO_4_∙12 H_2_O, 9.0; KH_2_PO_4_, 1.5; MgSO_4_∙7 H_2_O, 0.2; NH_4_Cl 1.0; CaCl_2_∙2 H_2_O, 0.02; Fe^III^NH_4_citrate, 0.0012; yeast extract, 0.5 with 20 g/L glycerol as sole carbon source. The MSM included 1 mL/L microelements solution (MES), which contains (g/L): EDTA, 50.0; FeCl_3_∙6 H_2_O, 13.8; ZnCl_2_, 0.84; CuCl_2_∙2 H_2_O, 0.13; CoCl_2_∙6 H_2_O, 0.1; MnCl_2_∙6 H_2_O, 0.016; H_3_BO_3_, 0.1; all these compounds were dissolved in distilled water. The cultivation was carried out for 48 h at 50 °C with shaking at 180 rpm. After cultivation, the fresh culture was used to inoculate fresh MSM; this second cultivation was carried out for 48 h at 50 °C with shaking at 180 rpm. After the second cultivation, the original isolation protocol was used to isolate the PHA-producing bacteria. The isolation protocol is based on the protective role of PHA against osmotic challenge. During the isolation, the mixed bacterial consortium obtained after the second cultivation is exposed to hyperosmotic challenge induced by solution of NaCl at a concentration of 100 g/L; subsequently, the culture is exposed to sudden hypotonic shock introduced by transfer of the culture to distilled water. Such a rapid fluctuation of osmotic pressure favors survival of PHA-producing bacteria, which are plated on solidified media, and PHA positive colonies are identified by infrared spectroscopy. The principle of the isolation procedure is depicted at Figure 1 and it was described previously [16].

### 2.2. Identification and Metabolic and Morphological Characterization of the Isolate

The isolate was identified by sequencing the *16S* rRNA gene. Cells were lysed by heating to extract DNA. A small amount of culture from Petri dishes was added to the lyses buffer (5 mM TrisHCl, pH 8.5) and heated to 95 °C for 10 min. After heating, lysed cells were centrifuged, and the supernatant was used as a DNA template. Polymerase chain reaction (PCR) for detection of bacterial DNA and taxonomy (*16S* rRNA) was performed using the forward primer 16S-F (5-′AAGAGTTTGATCCTGGCTCAG-3′) and the reverse primer 16S-R (5′-GGTTACCTTGTTACGACTT-3′). The PCR mixture consisted of 12.5 µL One TaqTM Hot Start 2X Master Mix with Standard Buffer, 2.6 µL MgCl_2_, 2 µL DNA template, 0.2 µM of each primer; this solution was added to 25 µL H_2_O, and the resulting PCR mixture was heated at 94 °C for 30 s. The PCR cycle consisted of three steps: denaturation for 30 s at 94 °C, annealing for 30 s at 55 °C and extension for 90 s at 68 °C. This cycle was repeated 30 times, the last extension step was extended for 5 min at 68 °C. The PCR amplicon was detected by 2% agarose gel electrophoresis with 1x TBE. PCR products of 16S rRNA gene were purified using the NucleoSpin Gel and PCR Clean-up kit and sent for commercial sequencing (SEQme, Dobris, Czech Republic). The taxonomic classification was evaluated using the blast NCBI database (https://blast.ncbi.nlm.nih.gov/Blast.cgi).

The standard set of physiological and biochemical tests such as shape and color of colonies, morphology of cells and spores, capability of hydrolysis of gelatin, urea, lecithin, starch, Tween 80, esculine, tyrosine and DNA, production catalase, arginine dehydrogenase and acid formation from various carbohydrates, spore formation, growth in presence of 10% and 70% NaCl, growth at various temperatures in range of 30–60 °C or hemolysis were performed at Czech Collection of Microorganisms according to procedures described in [17]. The isolate was also commercially identified using Biolog MicroStation system at Czech Collection of Microorganisms (CCM).

The morphology of cells was in more details investigated by Transmission Electron Microscopy (TEM) analysis using Microscope JEOL 1010 (JEOL, Pleasanton, USA) as described previously [18]. The high-pressure-freezing was performed using the instrument EM ICE (Leica Microsystems, Vienna, Austria) on the 3 mm aluminum carriers.

### 2.3. Production of PHA in Shaking Flasks by Aneurinibacillus sp. H1

To generate the inoculum, the culture was grown in complex media Nutrient Broth (10 g/L beef extract, 10 g/L peptone, 5 g/L NaCl) at 45 or 50 °C with shaking at 190 rpm. For production of PHA, mineral salt medium (MSM) was used consisting of: Na_2_HPO_4_∙12 H_2_O, 9.0 g/L; KH_2_PO_4_, 1.5 g/L; MgSO_4_∙7 H_2_O, 0.2 g/L; NH_4_NO_3_ 1.0 g/L; CaCl_2_∙2 H_2_O, 0.02 g/L; Fe^III^NH_4_citrate, 0.0012 g/L; Tryptone 0.5 g/L with 1 mL/L of MES (composition see 2.1). Various carbon sources (glycerol, glucose, sucrose, mannose, galactose, fructose, lactose, 1,4-butanediol, etc.) were usually applied at a concentration of 20 g/L if not stated otherwise. The inoculum ratio was 10 vol. %. Production cultivations were carried out for 72 h at selected temperature (usually 45 °C if not stated otherwise) under constant shaking of 180 rpm. All the cultivations were performed in duplicate. After cultivation, bacterial cells were harvested by centrifugation (6000× *g*, 5 min). Biomass was determined gravimetrically as the cell dry mass (CDM), and the amount and monomer composition of PHA in CDM was analyzed by Gas Chromatography as reported previously [19].

Among the tested substrates, we also included waste glycerol, which was obtained from the oil mill company Victoria Oil, Šid, Serbia. This side product of biodiesel production was previously analyzed on GC/FID as a part of regular control of biodiesel quality. It was found that the composition of waste glycerol mainly consisted of glycerol (83.72 *w*/*w* %), water (6.77 *w*/*w* %), ash/NaCl (6.5 *w*/*w* %) and other organic matter (1.58 *w*/*w* %). The pH value of waste glycerol was 6.77.

## 3. Results and Discussion

### 3.1. Taxonomic, Metabolic and Morphological Description the Isolate

Since we observed that among all isolates obtained from compost the strain designated as H1 reveals the most promising PHA production capacity, the isolate was phenotypically characterized by Czech Collection of Microorganisms. The cells are Gram-positive, and grow individually. **Positive tests**: spore formation; catalase; urease; anaerobic growth; hydrolysis of gelatin, ONPG and lecithin; acids produced from glucose, xylose and lactose; growth in presence of 7% NaCl; growth at 60 °C; hemolysis. **Negative tests**: starch hydrolysis, hydrolysis of: Tween 80, esculin, tyrosine and DNA, acetoin; reduction of nitrates; acids produced from mannitol, cellobiose, fructose and inositol; growth in presence of 10% NaCl, growth at 30 °C and 65 °C. Further, the isolate was also identified using the BIOLOG system, according to results of phenotype characterization and BIOLOG analysis, the strain was identified as *Aneurinibacillus* sp. H1 without possible reliable closer taxonomical classification. Further, the isolate was also identified by partial sequencing of *16S* rRNA gene, the sequence is available at MT112889; according to comparison of *16S* rRNA sequences available in BLAST database, the strain has high similarity to *Aneurinibacillus thermoaerophilus* strain DSM 10154 (99.91%), *Aneurinibacillus thermoaerophilus* strain L420-91 (99.83%), and *Aneurinibacillus sediminis* strain 1-10M-8-7-50 (96.47%).

The fact that the isolate is a Gram-positive bacterium can be considered being very beneficial. Most of the currently used PHA-producing strains are Gram-negative bacteria such as *Cupriavidus necator* [20,21], transgenic *Escherichia coli* [22] or members of the genus *Halomonas* [23,24]. Actually, one of the major obstacles preventing application of PHA in high-value applications such as health care, cosmetics or medicine is the fact that the polymers isolated from Gram-negative strains are heavily contaminated by lipopolysaccharides (LPS), a group of endotoxins produced by Gram-negative bacteria as important component of their outer cell membrane. LPS are co-isolated with PHA during down-stream processing and might cause severe immunological response making PHA biopolymers extremely unsuitable in medical uses [25].

Members of the genus *Aneurinibacillus* are mostly thermophilic or thermotolerant and, of course, they are taxonomically closely related to the genus *Bacillus*, which is well known for its capability of PHA biosynthesis [26,27,28]. Actually, the genus *Aneurinibacillus* was set aside from genus *Bacillus* in 1996 [29]. It should be pointed out that PHA production capacity was already described for thermophilic *Aneurinibacillus* sp. XH2 isolated from an oilfield in China [30]; hence, it seems that PHA biosynthesis is also ubiquitous among members of *Aneurinibacillus*.

**Figure 2:**
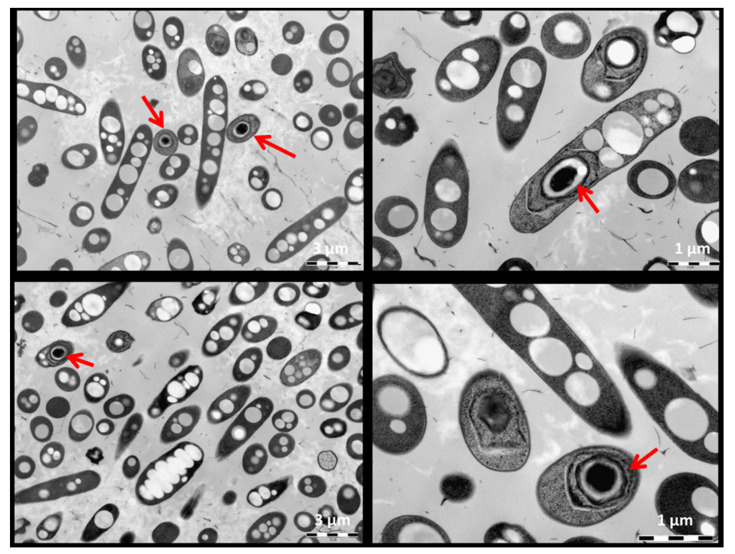
Morphology of the cells of *Aneurinibacillus* sp. H1 observed by TEM; the red arrows show endospores in bacterial cells.

The morphology of the cells of *Aneurinibacillus* sp. H1 grown for 72 h in mineral medium with glycerol was investigated by TEM, the results are shown in Figure 2. The cells contain 8–15 PHA granules, which are not localized in particular parts of the cells but are rather randomly distributed in the cellular space. Actually, size of the cells, localization and diameters of the granules are very similar to those reported for typical well-studied PHA producers such as *C. necator* H16 [31] or *Halomonas halophila* [32]. Apart from PHA granules, in some cells presence of spores in the central part of the cells can be observed as well (marked by red arrows). Generally, sporulation is not considered being a positive feature of the strains used in industrial biotechnology, since sporulation is associated with loss of desirable metabolic activity, and it was also reported that sporulation in *Bacillus* species is associated with mobilization of PHA storage [33]. From the economic point of view, spore formation depletes carbon sources dedicated to PHA biosynthesis. Nevertheless, despite the fact that cultivation was performed for a relatively long time (72 h), the number of sporulating cells is very low (below 10%), and PHA granules can be clearly seen also in spore forming cells, which indicates that sporulation should not be a major drawback preventing utilization of the isolate *Aneurinibacillus* sp. H1 in industrial production of PHA. However, gaining non-sporulating mutants of the strain would be very desirable. Further experiments could also clarify if shifting process parameters (e.g., pH-value) could be a tool to completely prevent spore formation, as it was previously demonstrated for PHA-accumulating *Bacillus cereus* SPV cultures, which at lower pH-value readily accumulated PHA, but completely stopped sporulation, thus drastically increasing the substrate-to-PHA yield [34].

### 3.2. Production of PHA on Various Carbon and Nitrogen Substrates

Further, the production capacity of the isolated strain *Aneurinibacillus* sp. H1 on various carbon substrates was evaluated; results are shown in Table 1. Obviously, the highest biomass growth and PHA titers were achieved on glycerol, since the bacterial strain was capable of reaching a CDM of 2.19 g/L, and the PHA content in CDM amounted to 45.95%. Since glycerol was used as the sole carbon substrate during isolation of the strain, it is not surprising that this strain gained high productivity on this substrate by adaptation. The second-best results, namely a CDM of 2.00 g/L and a PHA content in CDM of 27.74%, were obtained when glucose was used. In all the other tested carbohydrate substrates, CDM values as well as PHA contents were very low. It should be mentioned that, not surprisingly, on all the tested substrates the bacterial culture produced homopolymer P(3HB). Further, the culture is not capable of utilizing lipid substrates, since practically no biomass growth was observed on waste frying oil when used as a sole carbon source.

The fact that glycerol is the most preferred substrate by *Aneurinibacillus* sp. H1 can be considered as positive since waste glycerol is currently produced at steadily increasing quantity as a waste stream of biodiesel production [35]. Glycerol stemming from biodiesel fabrication contains numerous microbial inhibitors such as free fatty acids, methanol and residues of metal catalyst, which complicates its utilization as a substrate in microbial biotechnology. Nevertheless, there are reports on PHA production from waste glycerol employing various mesophilic microbes such as *Bacillus cereus* [36], *Cupriavidus necator* [37], *Burholderia cepacia* [38] or the extremely halophilic archaeon *Haloferax mediterranei* [39]; moreover, there are also some reports on conversion of glycerol into PHA by thermophiles, in particular by *Chelatococcus* sp. isolated from an aerobic organic waste treatment plant in Germany [13], and also by *Caldimonas manganoxidans* [40]. To evaluate the potential of the isolate *Aneurinibacillus* sp. H1 for PHA production from waste glycerol and to estimate robustness of the strain against inhibitors contained in real waste glycerol, we performed an experiment in which PHA production on pure and waste glycerol was assessed at various initial concentrations of both carbon substrates; the results are shown in Table 2. In both waste and pure glycerol, the highest P(3HB) titers were gained at initial substrate concentration of 20 g/L, the better values were, in accordance with our expectations, obtained with pure glycerol. However, *Aneurinibacillus* sp. H1 demonstrated expedient robustness against inhibitors present in waste glycerol, since product titers obtained on waste glycerol were only about 20% lower than on pure glycerol (1.70 g/L in pure and 1.42 g/L in waste glycerol), and polymer contents in biomass were very similar (51.97 and 49.45% of CDM for pure and waste glycerol, respectively).

### 3.3. Influence of Temperature on Production of PHA

We have also investigated the influence of the cultivation temperature on PHA production by *Aneurinibacillus* sp. H1. Cultivations were performed at temperatures in the range of 45–65 °C (Table 3.). The highest CDM concentration, PHA content and, therefore, also PHA titers were obtained at 45 °C; hence, this temperature can be considered being the optimal cultivation temperature. Further increase in cultivation temperature inhibited growth of the strain as well as PHA biosynthesis; nevertheless, the strain was capable of growing and synthesizing PHA even at 60 and 65 °C. Xiao et al. isolated *Aneurinibacillus* sp. designated as strain XH2 from the Gudao oilfield in China; optimal growth and PHA production temperature for this strain was reported as 55 °C, nevertheless, maximal PHA titers were gained at optimal conditions were only 0.268 g/L [30], which is substantially less than achieved with our isolate *Aneurinibacillus* sp. H1, since the maximal P(3HB) titer gained by our strain in flasks experiment was 2.03 g/L, almost one order higher, indicating the auspicious biotechnological potential of our isolate. 

The optimal cultivation temperature of 45 °C is most likely not high enough to eliminate the risk of contamination by ubiquitous thermotolerant microorganisms and, therefore, the biotechnological production process probably cannot be operated under economically and energetically attractive nonsterile open fermentation conditions. Nevertheless, despite this fact the industrial process could be energetically favorable since energy and water demands for cooling would be very low when cultivation would be performed at 45 °C as compared to standard mesophilic cultivation setups. Further, since 45 °C is not a very high temperature, energy demands on heating are not enormous and, moreover, it is likely that substantial amounts of heat needed to maintain the temperature at 45 °C would be generated by the metabolism of the microbes and also by the stirring system of the bioreactor, thus, the system could be partially operated as “self-heating system”, as described before [41]. Last but not least, since oxygen solubility negatively correlates with temperature, rational aeration of the bioreactor would be feasible. Nevertheless, these technological presumptions must be evaluated during forthcoming scale-up experiments, which are, nevertheless, above scope of this work focused on characterization of the novel thermophilic PHA producer.

### 3.4. Biosynthesis of PHA Copolymers and Terpolymer

In a subsequent experiment, we looked into the capability of the *Aneurinibacillus* sp. H1 to incorporate 3HV and 4HB into the polymer structure by using structurally related precursor compounds; results are shown in Table 4. The 3HV precursors (levulinate, propionate, n-propanol, valerate) were applied at a concentration of 2 g/L along with glycerol (20 g/L), which was used as the main carbon substrate. Application of all the tested precursors partially inhibited growth of the bacterial culture, which is not surprising since all of them are well known microbial inhibitors. Among tested substrates, only application of levulinate did not result in production of P(3HB-*co*-3HV) copolymers, while using all the other 3HV precursors, the bacterial culture was able to incorporate 3HV into the polymer chain. Valerate seems to be the most suiTable 3HV precursor since its application resulted in very high 3HV content (66.62 mol %) in P(3HB-*co*-3HV) copolymer, and its inhibitory effect on the growth of microbial culture was the lowest among the tested precursors.

Based on our experience, we utilized a different cultivation approach to gain copolymers containing 4HB subunits. Selected 4HB precursors (1,6-hexanediol, γ-butyrolactone and 1,4-butanediol) were applied as the sole carbon sources at a concentration of 8 g/L. The bacterial culture was not capable of readily assimilating the precursor with 6 carbon atoms (1,6-hexanediol); hence, only a very low amount of PHA was obtained on this compound, and the polymer contained no 4HB subunits. Oppositely, precursors containing 4 carbon atoms were assimilated by the bacterial culture much more efficiently and their use resulted in very high PHA contents in cells (85.84 and 75.48% of CDM for GBL and 1,4-butanediol, respectively) and also in very high 4HB portion in copolymers (63.35 and 79.91 mol % for GBL and 1,4-butanediol, respectively).

Further, we also attempted production of PHA terpolymer consisting of 3HB, 3HV and 4HB subunits. In this experiment, valerate (2 g/L) was used since it was identified as the most promising 3HV precursor, while 1,4-butanediol (4 g/L) was utilized as the most suiTable 4HB precursor. This cultivation strategy resulted in production of a very interesting terpolymer consisting of 33.13 mol % of 3HV, 54.18 mol % of 4HB and 12.69 mol % of 3HB.

These experiments proved the extraordinary capacity of *Aneurinibacillus* sp. H1 to biosynthesize polymers containing 3HV and/or 4HB subunits, which generally reveal superior mechanical properties compared to P(3HB) homopolymer. Especially the capacity of the strain to incorporate high portions of 4HB subunits into the polymer chain is very promising since the increased 4HB content in polymer is usually connected with improved flexibility as the crystallinity of the polymer is affected [42]. Capabilities to synthesize materials with so high 4HB fraction, reaching high intracellular PHA content and reasonable PHA titers are rare among PHA producers. Only few microorganisms are capable of production of PHA with 4HB fraction higher than 20–30 mol %. For instance, Huong et al. employed *Cupriavidus* sp. USMAA1020 for production of P(3HB-*co*-4HB) copolymer and 4HB fractions in polymer varying between 7 and 70 mol % depending on the applied cultivation conditions; nevertheless, increase of the 4HB fraction was accompanied by lowering of PHA content in bacterial cells, reduced biomass growth and lower product titers. When PHA with the highest 4HB portion of 70 mol % was produced, PHA content in CDM achieved only 13%, and the PHA concentration was only 0.3 g/L [43]. Similarly, *Comomonas acidovorans* was capable to synthesize P(3HB-*co*-4HB) copolymers with 4HB fractions up to 96 mol % when pure 4-hydroxybutyric acid was used as substrate; nevertheless, the PHA fraction in CDM reached only 25% [44]. Hence, it seems that isolate *Aneurinibacillus* sp. H1 is a very promising candidate for production of interesting PHA copolymers with tailored monomer composition and material properties. Apart from P(3HB-*co*-4HB) copolymers, it is also capable of production of P(3HB-*co*-3HV-*co*-4HB) terpolymers with high 3HV and 4HB fraction. Production of P(3HB-*co*-3HV-*co*-4HB) terpolymers was recently reported employing *Cupriavidus* sp. DSM 19379, nevertheless, to induce efficient terpolymer production, a sophisticated two-stage fermentation strategy was needed [45]. On the contrary, our isolate *Aneurinibacillus* sp. H1 produced desirable terpolymers in a simple single stage cultivation set up, which is a very positive feature. Further, it should be pointed out that, to our best knowledge, this is the first report on efficient production of PHA copolymers and terpolymers containing 4HB subunits by a thermophilic bacterium.

Since the ability to produce P(3HB-*co*-4HB) copolymer from 1,4-butanediol was extraordinary and it deserved further attention, we have tested a potential strategy how to further improve productivity and gain even higher product titers. At first, we optimized the initial concentration of 1,4-butanediol, the results are shown in Table 5. Decrease in initial concentration of the substrate from originally used concentration of 8 g/L increased biomass growth. According to our results, the optimal initial concentration of 1,4-butanediol is 4 g/L; at this concentration, biomass growth as well as PHA titer are enhanced substantially compared to the previous experiment and, surprisingly, the portion of 4HB in copolymer reached even 90 mol %, which further confirmed the promising potential of the bacterium. Increase in substrate concentration above 4 g/L resulted in inhibited growth and reduced PHA yields. 

To further enhance productivity, we decided to apply also glycerol along with 1,4-butanediol. The purpose of this step was to use glycerol as a co-substrate, which is efficiently utilized by the culture to support growth of the strain and, therefore, also final product titers. Thus, 1,4-butanediol was applied at an optimal concentration of 4 g/L, and glycerol was added at various concentration levels from 2 to 20 g/L; results are shown in Table 5. According to our expectation, introduction of glycerol enhanced growth of the bacterium, CDM values obtained with a mixture of 1,4-butanediol and glycerol are substantially higher than with 1,4-butanediol only. Results indicate that the optimal concentration of additional glycerol is 2 or 4 g/L, since these conditions resulted in high biomass growth (about 2.7 g/L), high PHA content in biomass (more than 65% of CDM), and high portion of 4HB in copolymer (83.56 and 74.43 mol % for 2 and 4 g/L glycerol, respectively). Generally, manipulating the concentration ratios of 1,4-butanediol to glycerol seems to be a simple and rational strategy how to control 4HB content in the polymer.

## 4. Conclusions

A recently established isolation protocol was utilized to gain new thermophilic PHA producers. Among the isolates tested, isolate designated as H1 turned out to be the most promising strain in terms of PHA production capability. The isolate was further taxonomically classified as a member of the genus *Aneurinibacillus*. According to subsequent experiments, it seems that *Aneurinibacillus* sp. H1 is an interesting thermophilic Gram-positive PHA accumulating bacterium with extraordinary ability to synthesize PHA copolymers and terpolymers containing high molar fractions of 3HV and 4HB subunits when valerate and/or 1,4-butanediol are used as 3HV and 4HB precursors, respectively. In considering all of the positive aspects of PHA production employing thermophiles, our isolate seems to be a highly intriguing potential candidate for PHA production on an industrial scale; therefore, the organism was deposited in Czech Collection of Microorganisms as patent culture CCM 8960. It should be also pointed out that the PHA materials produced by *Aneurinibacillus* sp. H1 were in depth characterized and the results have been submitted in an accompanying paper. Further experiments will be focused on optimization of PHA production under controlled conditions and its scale-up to laboratory bioreactors. In addition, we plan to sequence the entire genome of the bacterium to identify potential targets for genetic improvement of the strain.

## Figures and Tables

**Table 1:** Production of PHA on various carbon sources.

Substrate	CDM [g/L]	P(3HB) [% per CDM]	P(3HB) [g/L]
Sucrose	0.41 ± 0.10	16.52 ± 0.31	0.07 ± 0.02
Mannose	0.19 ± 0.08	14.85 ± 0.05	0.03 ± 0.01
Galactose	0.22 ± 0.04	8.94 ± 0.13	0.02 ± 0.00
Glucose	2.00 ± 0.36	27.74 ± 0.18	0.55 ± 0.10
Fructose	0.24 ± 0.01	14.32 ± 0.21	0.03 ± 0.00
Lactose	0.20 ± 0.01	9.06 ± 0.20	0.02 ± 0.00
Glycerol	2.19 ± 0.07	45.95 ± 0.10	1.00 ± 0.04
WFO ^1^	0.01 ± 0.00	*n.d.*	*n.d.*

^1^ WFO stands for Waste Frying Oils. n.d.: not detected.

**Table 2:** Comparison of PHA biosynthesis on pure glycerol and waste glycerol from biodiesel production.

Substrate	Concentration [g/L]	Biomass [g/L]	P(3HB) [% per CDM]	P(3HB) [g/L]
**Glycerol**	10	1.60 ± 0.50	13.00 ± 1.20	0.21 ± 0.03
	20	3.27 ± 0.13	51.97 ± 2.28	1.70 ± 0.11
	30	2.72 ± 0.07	53.40 ± 4.82	1.45 ± 0.19
**Waste/crude Glycerol**	10	1.08 ± 0.08	10.78 ± 1.33	0.12 ± 0.02
	20	2.88 ± 0.13	49.45 ± 0.65	1.42 ± 0.03
	30	1.67 ± 0.32	46.10 ± 5.55	0.77 ± 0.13

**Table 3:** Effect of cultivation temperature on PHA yields obtained by *Aneurinibacillus* sp. H1.

Temperature [°C]	Biomass [g/L]	P(3HB) [% per CDM]	P(3HB) [g/L]
35	0.17 ± 0.01	30.20 ± 0.01	0.05 ± 0.01
40	2.66 ± 0.14	32.12 ± 4.49	0.92 ± 0.08
45	3.68 ± 0.63	55.31 ± 5.81	2.03 ± 0.41
50	3.23 ± 0.03	46.01 ± 3.55	1.49 ± 0.37
55	1.46 ± 0.05	50.18 ± 1.35	0.73 ± 0.03
60	0.80 ± 0.01	30.60 ± 1.35	0.24 ± 0.01
65	0.89 ± 0.06	32.66 ± 0.24	0.29 ± 0.02

**Table 4:** Production of PHA copolymers and terpolymer using various precursors of 3HV and 4HB.

Desired Monomer	Precursor	Biomass [g/L]	PHA [% per CDM]	PHA [g/L]	3HV [mol %]	4HB [mol %]	3HB [mol %]
3HV ^1^	**Levulinate**	1.08 ± 0.06	36.70 ± 0.72	0.39 ± 0.02	*n.d.*	*n.d.*	100.00 ± 0.00
	**Propionate**	1.11 ± 0.04	28.15 ± 0.27	0.31 ± 0.01	32.10 ± 0.02	*n.d.*	67.90 ± 0.02
	**Propanol**	1.47 ± 0.04	45.11 ± 0.05	0.66 ± 0.02	3.66 ± 0.04	*n.d.*	96.34 ± 0.04
	**Valerate**	1.90 ± 0.02	36.29 ± 0.01	0.69 ± 0.01	66.62 ± 0.83	*n.d.*	33.38 ± 0.83
4HB ^2^	**1,6-hexandiol**	0.37 ± 0.08	9.08 ± 0.31	0.03 ± 0.01	*n.d.*	*n.d.*	100 ± 0.00
	**γ-butyrolactone**	0.52 ± 0.00	85.84 ± 1.99	0.45 ± 0.01	*n.d.*	63.35 ± 3.27	36.65 ± 3.27
	**1,4-butandiol**	1.02 ± 0.04	75.48 ± 2.36	0.77 ± 0.04	*n.d.*	79.91 ± 1.84	20.09 ± 1.84
3HV + 4HB ^3^	**Valerate + 1,4-butanediol**	1.44 ± 0.06	40.27 ± 2.08	0.58 ± 0.03	33.13 ± 1.03	54.18 ± 1.23	12.69 ± 2.26

^1^ 3HV precursors were applied at the beginning of cultivation at concentration of 2 g/L. 20 g/L of glycerol was used as the main carbon source. ^2^ 4HB precursors were applied at the beginning of cultivations as the sole carbon sources at concentration of 20 g/L. ^3^ Terpolymer production was achieved by applying 1,4–butanediol (4 g/L) and valerate (2 g/L) at the beginning of cultivation, glycerol was not added. *n.d.*: not detected.

**Table 5:** Effect of initial concentration of 1,4-butanediol and glycerol on production of P(3HB-*co*-4HB) by *Aneurinibacillus* sp. H1.

1,4-BD [g/L]	Glycerol [g/L]	Biomass [g/L]	PHA in CDM [%]	PHA [g/L]	4HB in PHA [mol %]
3	0	1.22 ± 0.11	50.14 ± 2.65	0.61 ± 0.07	90.56 ± 0.70
4	0	1.67 ± 0.02	54.78 ± 1.21	0.91 ± 0.02	90.89 ± 1.19
5	0	1.60 ± 0.23	45.73 ± 0.65	0.73 ± 0.11	88.31 ± 0.29
6	0	1.23 ± 0.15	56.32 ± 0.56	0.69 ± 0.08	92.81 ± 0.04
7	0	1.13 ± 0.01	51.80 ± 0.71	0.58 ± 0.01	88.02 ± 0.48
8	0	0.96 ± 0.00	44.00 ± 2.76	0.42 ± 0.03	86.87 ± 1.32
12	0	0.72 ± 0.06	43.90 ± 2.28	0.32 ± 0.03	83.61 ± 0.94
16	0	0.56 ± 0.05	43.38 ± 1.26	0.24 ± 0.02	85.99 ± 4.40
4	2	2.69 ± 0.15	68.25 ± 0.62	1.83 ± 0.10	83.56 ± 1.48
4	4	2.79 ± 0.03	65.23 ± 2.64	1.82 ± 0.08	74.43 ± 1.70
4	6	2.28 ± 0.26	50.37 ± 0.87	1.15 ± 0.13	42.45 ± 7.88
4	8	2.43 ± 0.01	40.74 ± 3.35	0.99 ± 0.08	36.42 ± 1.63
4	20	2.56 ± 0.11	44.69 ± 1.23	1.14 ± 0.06	4.59 ± 0.01
